# Design of Flexible Film-Forming Polydopamine/Polypyrrole/Nanodiamond Hierarchical Structure for Broadband Microwave Absorption

**DOI:** 10.3390/polym14102014

**Published:** 2022-05-15

**Authors:** Ruoxuan Huang, Yan Zhang, Zhiyong Zhang, Guangjun Gou, Xiangnan Chen

**Affiliations:** 1 College of Transportation Engineering, Dalian Maritime University, Dalian 116026, China; huan0237@ntu.edu.sg (R.H.); hrx0330@126.com (Y.Z.); zhiyongzhang74520@163.com (Z.Z.); 2 School of Materials Science and Engineering, Southwest University of Science and Technology, Mianyang 621010, China; hyb0509@gmail.com

**Keywords:** microwave absorption, film-forming, polydopamine, polypyrrole, nanodiamond composite

## Abstract

Microwave-absorbing materials are widely used in numerous fields, including the military, daily protection, etc. Currently, in addition to being lightweight and highly efficient, good film-forming processing characteristics and environmental stability are also required for the practical application of microwave-absorbing materials, which, in general, are difficult to make compatible. In this paper, a mulberry-like PDA/PPy/ND hierarchical structure was prepared by in situ polymerization. The hierarchical structure showed remarkably enhanced microwave absorption, as well as better flexible film-forming characteristics, thanks to the multiple roles PDA played in the system. The optimal RL peak for PDA/PPy/ND could reach −43.6 dB at 7.58 GHz, which is mainly attributed to the multiple dielectric loss paths and significantly improved impedance-matching characteristics. Furthermore, given the H-bond crosslink, the introduction of PDA also promoted the film formation and dispersion of PDA/PPy/ND in the PVA matrix, forming a water-resistant and flexible film. This work provides a referencing path for the design and practical applications of lightweight microwave-absorbing materials.

## 1. Introduction

Microwave absorbing materials (MAMs) will have great application prospects in the future [1,2]. In many fields, electromagnetic signals are inevitably used. Some of these signals need to be absorbed for purposes of stealth, and some need to be shielded to prevent signal interference, while others need to be dissipated to prevent harm to the human body [3,4,5]. A series of lightweight MAMs has been explored with excellent microwave-absorbing (MA) performances, such as conducting polymer [6,7], graphene [8,9], porous carbon [10,11], Maxene [12] and so on [13]. However, for practical applications, in addition to considering the improvement in MA performance, it was also necessary to ensure that the material had good machinability [14] in order to further construct subsequent MA devices or units.

Nanodiamond (ND), like other lightweight carbon nanomaterials, has excellent designable MA properties [15]. It has been found that the MA band, intensity and effective bandwidth can all be effectively regulated by controlling the surface hybridizations of ND [16,17]. In addition, ND possesses other special characteristics, such as good wear resistance, excellent stability, high hardness, etc. [18], which provides great application prospects for MAMs. In recent years, one of the most effective methods to realize broadband MA has been to construct hierarchical structures [19,20,21]. Hierarchical structures can bring synergistic electromagnetic loss mechanisms involving polarization relaxation loss, interface loss, structural resonance loss and multi-component coupling effects so as to improve MA performance. As a conducting polymer, polypyrrole (PPy) is often used in dielectric MAMs [17]. Thus, it was expected to further improve MA performance through the hierarchical structure design involved with PPy and ND.

Multi-component hybridization has also been proven to be able to optimize impedance matching [22,23,24,25]. Another way to optimize MA performance was to regulate the aggregation morphology of the microstructure [26]. Among them, dopamine (DA) had double-reactive sites, which could regulate the aggregation morphology of nanostructures [27], introducing multiple hybridizations and constructing hierarchical structures, which would possibly further improve electromagnetic (EM) characteristics. In addition, it has also been reported that DA can effectively enhance the dispersion of nanomaterials and could be used in film and hydrogel formation, based on the hydrogen-bond crosslink [28].

Thus, in this paper, polydopamine/polypyrrole/nanodiamond (PDA/PPy/ND) hierarchical structures were constructed to realize the compatibility of MA performance and film-forming machinability. It was found that a hierarchical mulberry-like PDA/PPy/ND hybrid could be synthesized by applying in situ polymerization, and it exhibited remarkably enhanced microwave absorption and better film-forming properties. The optimal reflection loss (RL) peak of PDA/PPy/ND could reach −43.6 dB at 7.58 GHz. Meanwhile, uniform flexible PDA/PPy/ND film could be fabricated using polyvinyl alcohol as a film-forming aid by using a simple solution-casting method. This work provides a referencing path for the design and even practical applications of lightweight MAMs.

## 2. Materials and Methods

### 2.1. Materials

The pyrrole, concentrated hydrochloric acid, ammonium persulfate and ethanol (95%) were all of analytical grade and supplied by Tianjin Kemi Chemical Reagent Co., Ltd. of Tianjin, China, Macklin, lnc of Shanghai, China, Sinopharm Chemical Reagent Co., Ltd. of Shanghai China, and Tianjin Fuyu Fine Chemical Co., Ltd. of Tianjin, China, respectively. ND (5–10 nm in diameter) was purchased from XF NANO, Inc. of Nanjing, China. The dopamine hydrochloride (DA, 98%) and polyvinyl alcohol (PVA 1788) were supplied by Macklin, Inc. Deionized water was prepared by ourselves in the laboratory.

### 2.2. Preparation of PDA/PPy/ND Hybrid

PDA/PPy/ND ternary hybrids were synthesized by applying in situ polymerization. All reactions were kept under 0–5 °C. The pyrrole concentration was controlled at 0.02 mol/L and the Py/APS/ND molar ratio at 1:1:1. First, 24 mg of ND and 18 mL of DA were added to 50 mL of HCl solution (Solution 1) and sonicated for 30 min. At the same time, 0.14 mL of pyrrole was added to 50 mL HCl solution (Solution 2) and stirred for 30 min. Then, Solution 1 and Solution 2 were mixed, and 0.456 g ammonium persulfate dissolved in 50 mL of HCl solution was dropped into the mixed solution and continuously stirred for 4 h. After being kept for 16 h, the product of PDA/PPy/ND was washed with deionized water and ethanol three times and then dried and collected.

### 2.3. Preparation of PDA/PPy/ND Film

The mass ratio of PVA to PDA/PPy/ND was controlled at 4:1. First, 7.5 g of PVA was added to 143 mL of distilled water and stirred at 95 °C for 1 h to form a PVA solution. Then, 1.875 g PDA/PPy/ND was added to the PVA solution and stirred for 1 h and then ultrasonicated for 1 h. Finally, 50 mL of the above mixed solution was poured into a PTFE mold with a diameter of 12 cm and placed in a 30 °C oven for 20 h to obtain a PDA/PPy/ND film.

### 2.4. Characterization

Field emission scanning electron microscopy (FESEM, FEI Inspect F50) and transmission electron microscopy (TEM, JEM-2100, JEOL) were carried out for morphology and structure analysis. An analysis of X-ray diffraction (XRD, Rigaku D/MAX-Ultima X-ray diffractometer, Cu Kα, 4°/min)) was used to characterize the orientation and ordered structure of the samples. Fourier-transform infrared spectroscopy (FTIR, Bruker TENSOR II spectrometer, with 4 cm−1 resolution) was used for the detailed confirmation of hybrid interactions of powder samples. Raman spectra were characterized on the LabRAM HR Evolution (Horiba Scientific, 514 nm, 400–4000 cm^−1^ with 2 cm^−1^ resolution). The electrical conductivities of samples were measured on an SZ82 digital instrument (four-probe method, at room temperature). The EM parameters were measured using a vector network analyzer (2–18 GHz, E5071 C, Agilent). The EM test samples were made into toroidal shapes (outer diameter of 7.0 mm, inner diameter of 3.04 mm and thickness below 5 mm, mass ratio of samples to wax = 3:7). Film thicknesses were measured using a spiral micrometer (SanLiang, Japan, 0–25 mm with 1 μm resolution).

## 3. Results and Discussion

**Figure 1:**
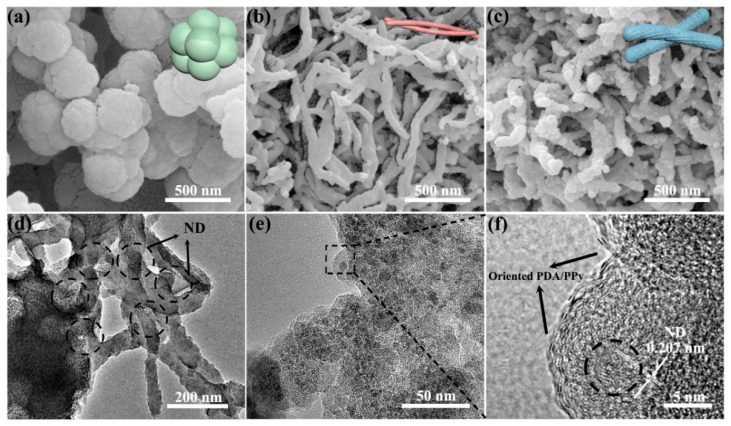
SEM images of (**a**) HCl-PPy, (**b**) PDA/PPy and (**c**) PDA/PPy/ND. The insert is the schematic for the related morphologies, (**d**,**e**) TEM images and (**f**) the HR-TEM image of PDA/PPy/ND.

Figure 1 shows the SEM images of HCl-PPy, PDA/PPy and PDA/PPy/ND. The overall morphological uniformity of the related samples can be found in supporting information Figure S1. It is notable that the morphologies changed significantly after adding PDA. HCl-PPy presented uniform spherical particles (Figure 1a and Figure S1a), while the morphologies obviously changed to long rods for PDA/PPy (Figure 1b and Figure S1b) and to hierarchical mulberry shapes for PDA/PPy/ND (Figure 1c and Figure S1c). Morphology changes could be mainly caused by two reasons. First, the possible hydrogen bonding might affect self-assembly driving forces during the polymerization of Py. Second, the unique surface-modification characteristics of DA [29] could lead to specific surface morphologies, which agreed well with our previous work involving PDA/PANi/ND [30]. The TEM images of PDA/PPy/ND can provide more detailed information about the hierarchical structure, as shown in Figure 1d–f. It was found out ND dispersed well in the mulberry-like matrix. Furthermore, in the local enlarged TEM image of the surface bulge (Figure 1e,f), ND with lattice fringes corresponding to the (111) plane (d = 0.207 nm) [31] can be found, accompanied by the local-oriented PDA/PPy onsite. This phenomenon indicated that ND was well preserved after polymerization, and ternary interfaces were formed with the partial orientation of the polymer along the surface of the ND.

**Figure 2:**
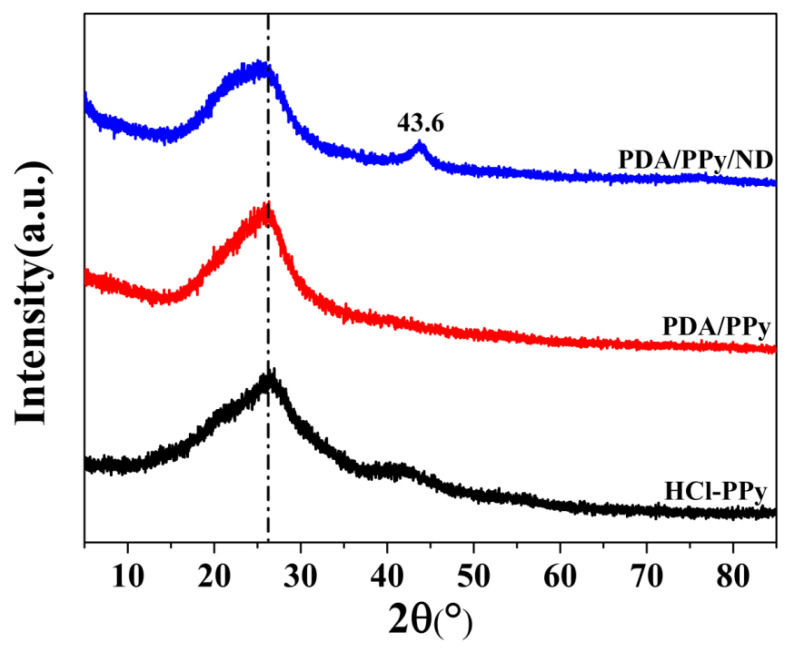
XRD patterns of HCl-Ppy, PDA/Ppy and PDA/Ppy/ND.

XRD patterns of PDA/Ppy/ND and the related samples are compared in Figure 2. For HCl-Ppy, the peak of amorphous Ppy can be found at 2θ = 25.8° [32], and in the XRD pattern of PDA/Ppy, the broad band at 2θ = 25.2° should be attributed to both the amorphous Ppy and PDA [33]. It is notable that the characteristic peak at 2θ = 43.6°, corresponding to the ND (111) plane [34], appeared in the XRD pattern of PDA/Ppy/ND, illustrating the complete preservation of ND crystals in the composites. Furthermore, for PDA/Ppy/ND, it can be seen that the amorphous polymer peak shifted to 2θ = 24.4°, with the peak shape changing significantly. Combined with SEM and TEM results, the generation of the hierarchical mulberry-like structure in PDA/Ppy/ND remarkably affected the orientation of the amorphous polymer.

**Figure 3:**
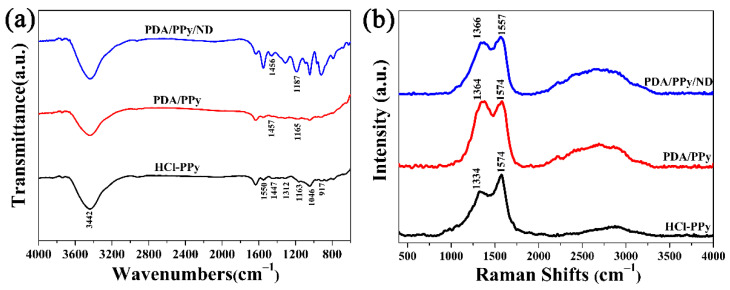
(**a**) FTIR spectra of HCl-Ppy, PDA/Ppy and PDA/Ppy/ND. (**b**) Raman spectra of HCl-Ppy, PDA/Ppy and PDA/Ppy/ND.

FTIR spectra were obtained to clarify the hybridization forms in PDA/Ppy/ND and the control samples, as shown in Figure 3a. In the FTIR spectra of HCl-Ppy, bands at 3442 (-OH and -NH stretching vibrations), 1550 (C=C stretching vibration in pyrrole ring), 1447 (C-C stretching vibration in pyrrole ring), 1312 and 1046 (=C-H in plane deformation), 1163 (C-N stretching vibration) and 917 (=C-H out-of-plane deformation) cm^−1^ could all be observed, corresponding to the functional groups of Ppy [35,36]. For both PDA/Ppy and PDA/Ppy/ND, the bands attributed to the C-C stretching in pyrrole rings presented obvious blue shifts (to 1457 cm^−1^ for PDA/Ppy and 1456 cm^−1^ for PDA/Ppy/ND), indicating π-π stacking between the benzene ring in PDA and pyrrole rings in Ppy [37]. In addition, the C-N stretching bands at 1163 cm^−1^ showed greater blue shifts to 1187 cm^−1^ for PDA/Ppy/ND, which may have resulted from the extra asymmetric polar hybridizations among PDA, Ppy and ND involved with the confinement effect of the ND surface [38], making C-N groups more stable. The multiple hybridizations in PDA/Ppy/ND were beneficial for improving dielectric dissipations.

The Raman spectra could be further evidence for multiple hybridizations, as shown in Figure 3b. The bands at 1334 and 1574 cm^−1^, corresponding to the pyrrole-ring stretching and C=C backbone stretching [39], showed obvious overlapping in both PDA/Ppy and PDA/Ppy/ND. In particular, the two bands for PDA/Ppy/ND shifted to 1366 and 1557 cm^−1^, confirming the hybridizations on Ppy [40]. Additionally, the Ppy-typical bands due to C-H out-of-plane deformation (932 cm^−1^) [41] both disappeared in the Raman spectra of PDA/Ppy and PDA/Ppy/ND, which is likely due to π-π stacking interactions, further limiting C-H deformations. Given the FTIR results, these phenomena further revealed π-π stacking and asymmetric polar hybridizations on the C-N groups in PDA/Ppy/ND.

**Figure 4:**
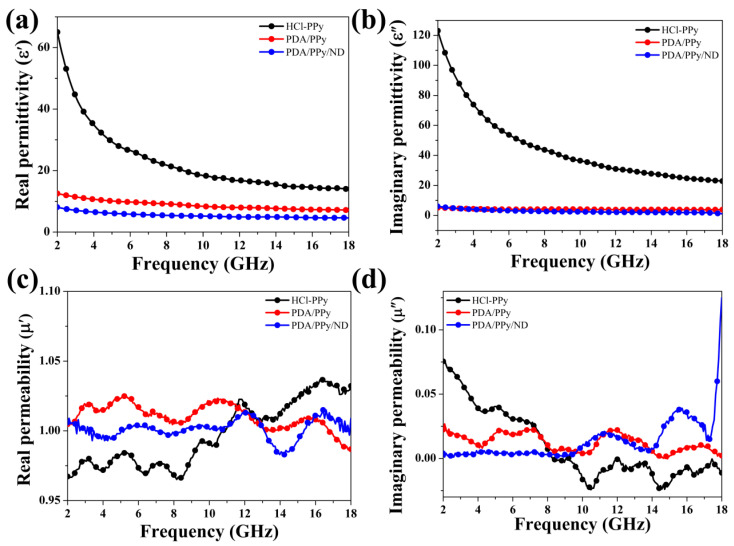
The measured EM parameters of the samples: (**a**) real permittivity, (**b**) imaginary permittivity, (**c**) real permeability and (**d**) imaginary permeability.

The EM parameters of the samples were characterized as shown in Figure 4 in order to investigate the MA properties of the samples. It was found that HCl-Ppy possessed the highest values of real permittivity (ε′) (Figure 4a) and imaginary permittivity (ε″) (Figure 4b). On the other hand, for PDA/Ppy and PDA/Ppy/ND, the values of ε′ and ε″ both showed obvious decreases (Figure 4a,b), which could lead to better synergy matching of the dielectric loss and magnetic loss [42]. The conductivities of the samples are also compared in Figure S2, illustrating obvious correlations with the value of permittivity. Actually, the dielectric loss of HCl-Ppy is mainly due to conductivity loss [43], while the dielectric loss of PDA/Ppy/ND should correlate to the polarization relaxation loss and interfacial relaxation loss [44] generated from the asymmetric polar hybridizations as well as the ternary interfaces [45], according to the above structural results. On the other hand, the values of real permeability (μ′) (Figure 4c) and imaginary permeability (μ″) (Figure 4d) were all close to that of air, indicating that there were almost no magnetic responses for all three samples. The dielectric loss tangent (tan δe) and magnetic loss tangent (tan δm) were also calculated to further compare dielectric and magnetic loss characteristics, as shown in Figure S3. The higher conductivity and permittivity could lead to poor impedance matching due to the great difference between dielectric loss and magnetic loss characteristics [46].

**Figure 5:**
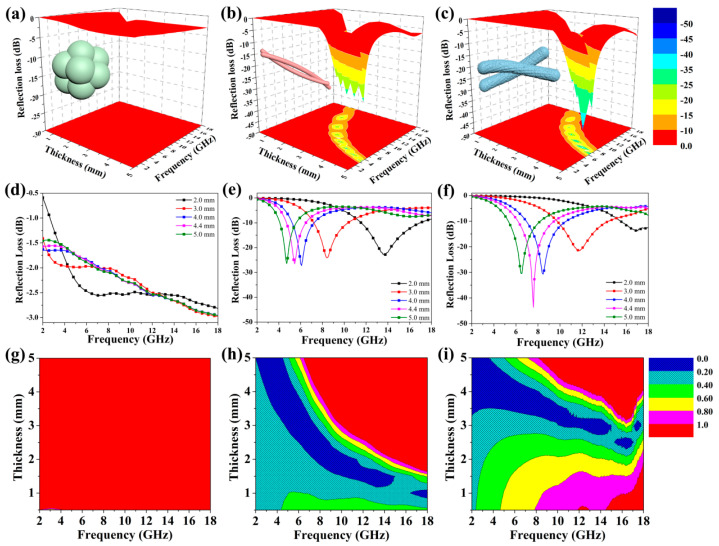
The calculated 3D RL map with a thickness of 0.5–5.0 mm for (**a**) HCl-PPy, (**b**) PDA/PPy and (**c**) PDA/Ppy/ND; the calculated RL value with certain thicknesses for (**d**) HCl-Ppy, (**e**) PDA/PPy and (**f**) PDA/PPy/ND; and the EM impedance matching degree Δ maps for (**g**) HCl-PPy, (**h**) PDA/PPy and (**i**) PDA/Ppy/ND. The inserts are the schematic for the related morphologies.

The microwave-absorbing properties were further compared, as shown in Figure 5. Reflection loss (RL) can be calculated based on the measured EM parameters according to the transmission line theory [47]:

RL = 20 log |(Z_in_ – Z_0_)/(Z_in_ + Z_0_)|, (1)

Z_in_ = Z_0_ [√ (μr⁄εr)] tan h [j (2 π f d⁄c) (√ (εr μr)], (2)

where Z_in_ respresents characteristic impedance, and Z_0_ respresents free space impedance. εr represents relative complex permittivity, and μr represents relative complex permeability. Variables d, c and f are the thickness, free space microwave velocity and microwave frequency, respectively.

Figure 5a–c show the calculated 3D RL map for the related samples with a thickness of 0.5–5.0 mm. The hierarchical PDA/Ppy/ND hybrids exhibited remarkably improved MA properties compared to those of HCl-Ppy and PDA/Ppy, with stronger optimal RL values and wider effective bandwidths. For PDA/Ppy/ND, the effective bandwidth reached 12.8 GHz, covering 5.2–18 GHz under a thickness of 1.8–5.0 mm. For PDA/Ppy, the effective bandwidth reached 15.2 GHz, covering 2.8–18 GHz under a thickness of 1.3–5.0 mm. In contrast, the RL of HCl-Ppy could not reach below −10 dB in the full band. Combined with the structural analysis, the introduction of the PDA and ND, as well as the corresponding morphology change, led to specific MA properties.

The RL results with certain thicknesses were further compared, as shown in Figure 5d–f. For HCl-Ppy, the optimal RL value could only reach −3.0 dB. The unitary dielectric loss form, which was conductivity loss, as well as the high conductivity and mismatched magnetic loss resulted in poor MA performance for HCl-Ppy. For PDA/Ppy, the optimal RL value reached −27.2 dB at 6.08 GHz (effective bandwidth = 2 GHz, covering 5.2–7.2 GHz), with a thickness of 4.0 mm. The improvement in MA performance for PDA/Ppy was because of the enhancement in dielectric polarization loss due to π-π interactions between PDA and Ppy [48]. Notably, the ternary PDA/Ppy/ND hierarchical structure presented the best MA properties. The optimal RL value of PDA/Ppy/ND achieved −43.6 dB at 7.58 GHz with a thickness of 4.4 mm (effective bandwidth = 3.3 GHz, covering 6.2–9.5 GHz). The further improvement of MA performance for PDA/Ppy/ND is obtained from multiple dielectric loss forms, which includes enhanced dielectric polarization relaxation losses caused by multiple polar hybridizations [49], extra interfacial relaxation losses due to the introduction of ND [50] and multiple reflection losses caused by the hierarchical structure [51]. The average diameter of the PDA/Ppy/ND hierarchical structure was above 500 nm, with the micron-scaled aggregate being in the same order of magnitude as the microwave wavelength (Figure 1c,d), which could lead to multiple reflections among the mulberry-like structures [52]. The MA properties of Ppy-related hybrids were compared, as shown in Table 1. It is notable that PDA/Ppy/ND could achieve similar or even stronger optimal RL values. More importantly, the EM response frequency band of PDA/Ppy/ND, covering 6.2–9.5 GHz, was different from most of the reported values in the literature.

In order to further clarify the essential reason for the differences in microwave-absorbing properties, the microwave impedance matching degrees ∆ were calculated according to the following delta function [53]:

∆ = |sin h^2^ (K f d) − M|, (3)

where K and M were calculated from the measured EM parameters according to the following formulas.

(4) $$K=\frac{4\pi \sqrt{\;\mu^{\prime }\;\varepsilon^{\prime }}}{c}\frac{\sin\left[\delta_{e}+\delta_{m}/2\right]}{\cos\delta_{e}\cos\delta_{m}},$$

(5) $$M=\left[4\;\mu^{\prime }\cos\delta_{e}\;\varepsilon^{\prime }\cos\delta_{m}\right]\cdot {\left[{\left(\;\mu^{\prime }\cos\delta_{e}-\;\varepsilon^{\prime }\cos\delta_{m}\right)}^{2}+{\left(\tan\frac{\delta_{m}-\delta_{e}}{2}\right)}^{2}\left(\;\mu^{\prime }\cos\delta_{e}+\;\varepsilon^{\prime }\cos\delta_{m}\right)\right]}^{-1},$$

As shown in Figure 5g–i, HCl-PPy displayed a poor microwave impedance matching degree, with all the values of ∆ being above 1.0. However, with the introduction of PDA, the impedance matching degree was significantly promoted due to a reduction in conductivity. Compared with PDA/pPy, the impedance matching degree of PDA/pPy/ND was further improved, which might have been due to the formation of the hierarchical gradient structure, i.e., the gradient impedance matching between the outer PDA/pPy and the inner polymer-coated ND, which encouraged microwaves entering and then dissipating in the hybrids [54].

**Figure 6:**
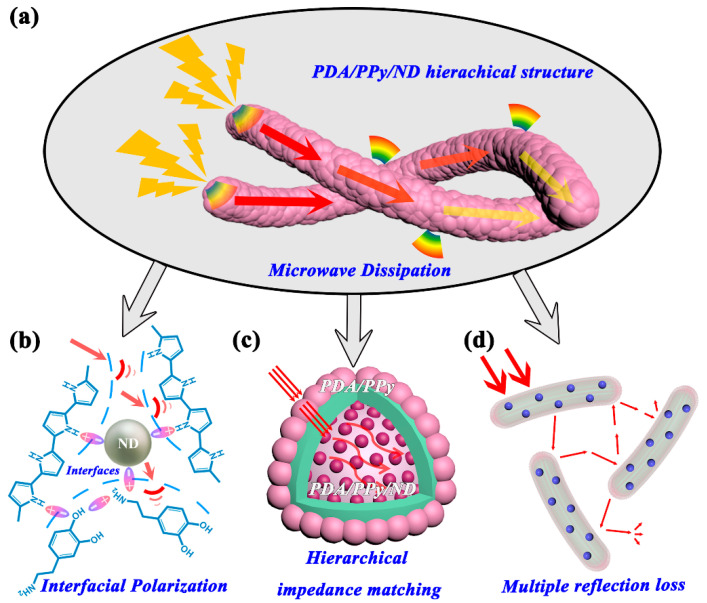
Schematic of the microwave-absorption mechanism for PDA/pPy/ND hierarchical structures. (**a**) the microwave dissipations in PDA/PPy/ND hierarchical structures, (**b**) the interfacial polarization losses in the hybrids, (**c**) the Hierarchical impedance matching between PDA/PPy and PDA/PPy/ND, (**d**) the multiple reflection loss in the hybrids.

Based on the structure and property analysis above, the MA mechanism for PDA/pPy/ND hierarchical structures is put forward, as shown in Figure 6. First, multiple hybridizations existed among PDA, pPy and ND, such as π-π stacking interactions and asymmetric polar hybridizations on the C-N groups. These hybridizations could lead to extra dipoles, acting as asymmetric polarization centers and generating more interfacial polarization relaxation losses. Second, the proper reduction in conductivity as well as the hierarchical gradient impedance matching between the outer PDA/pPy and the inner polymer-coated ND benefited microwave dissipation. Finally, the hierarchical structures being in the same order of magnitude as the microwave wavelength could introduce multiple reflection losses. Above all, the enhancement in microwave absorption should be mainly attributed to the multiple dielectric loss mechanisms and significantly improved impedance-matching characteristics.

**Figure 7:**
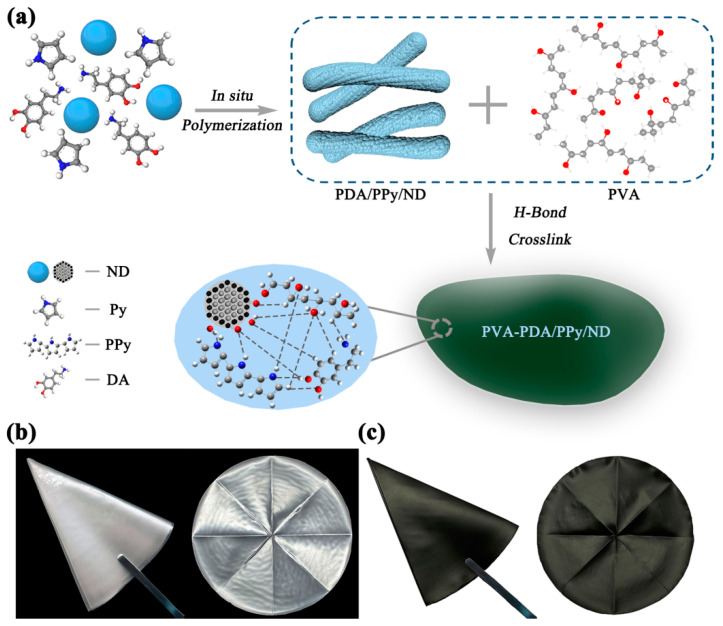
(**a**) Design diagram for PDA/pPy/ND film, using PVA as a film-forming aid and digital photos of (**b**) PVA film without adding PDA/pPy/ND; (**c**) PDA/pPy/ND film.

**Figure 8:**
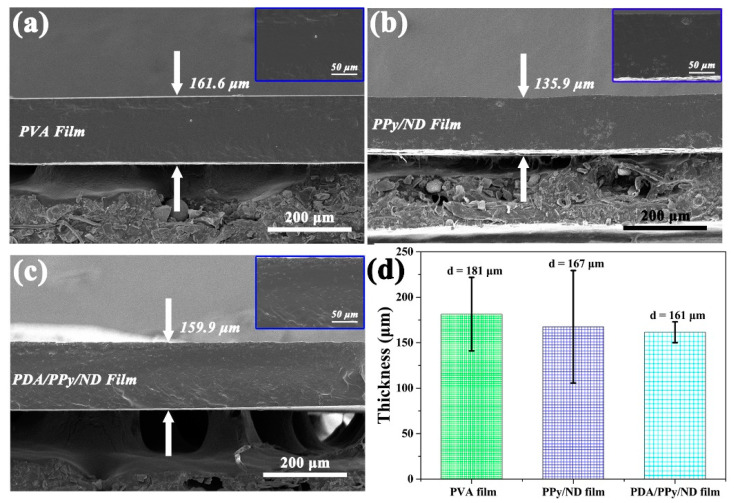
The SEM images of the film cross sections: (**a**) PVA film, (**b**) PPy/ND film and (**c**) PDA/PPy/ND film; the insert images in (**a**–**c**) were the related local enlarged SEM images, and (**d**) the film thicknesses measured by the spiral micrometer.

The film-forming property is very important for the practical applications of MAMs, for the functional powder must be dispersed into a resin or coating matrix in order to further obtain the actual MA functional device. In our study, PVA was used as an auxiliary agent to explore the film-forming characteristics of PDA/pPy/ND. As shown in Figure 7a, considering the H-bond among PDA, pPy, ND and PVA [55,56,57], it is possible to construct stable films based on the H-bond crosslink. It is notable that, as shown in Figure 7c, a flexible PDA/pPy/ND film can be obtained by applying a simple solution-casting method using PVA as a film-forming agent. The PDA/pPy/ND film showed better homogeneity compared with that of the pPy/ND film (Figure S4), which is likely due to the promoting effect of PDA for film formation and dispersion. Figure 8a–c provide SEM images for the cross sections of the related films. It was discovered that the fillers’ dispersion in PVA was significantly improved after adding PDA, as shown in the images of Figure 8a–c. Film thicknesses were also measured by the spiral micrometer, as shown in Figure 8d, which agreed well with the SEM results. The average film thicknesses for PVA, pPy/ND and PDA/pPy/ND were 181, 167 and 161 μm, respectively. Moreover, it is notable that the thickness for PDA/pPy/ND film became more uniform among the three samples based on the hydrogen bond network of PDA.

**Figure 9:**
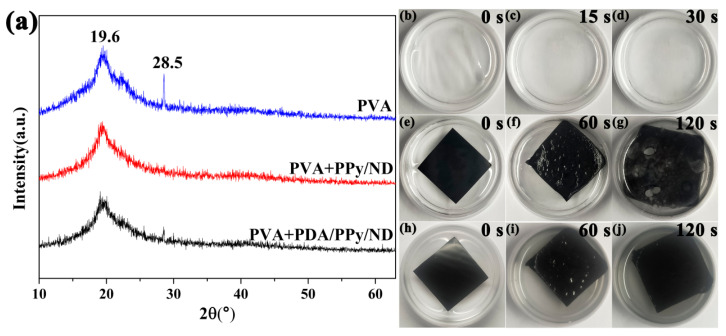
(**a**) The XRD patterns of the films, (**b**–**d**) the photographs of the PVA film after ultrasonication in water with corresponding times, (**e**–**g**) the photographs of the PPy/ND film after ultrasonication in water with corresponding times and (**h**–**j**) the photographs of the PDA/PPy/ND film after ultrasonication in water with corresponding times.

The XRD patterns of the films are shown in Figure 9a. Two obvious bands existed at 2θ = 19.6 and 28.5°, which are attributed to the semi-crystalline phase of the PVA [58]. It is notable that for the PPy/ND film, the peak at 2θ = 28.5° disappeared, indicating a decrease in PVA crystallinity, which could have resulted from the heterogeneous dispersion of PPy/ND in PVA. On the other hand, for PDA/PPy/ND, the peak at 2θ = 28.5° still existed, which further revealed the better dispersion of PDA/PPy/ND in the PVA matrix. The stabilities of the films toward water were also compared, as shown in Figure 9b–j. It is obvious that the water resistance of the PDA/PPy/ND film significantly improved. For the PPy/ND film, it began to decompose after sonification for 120 s (Figure 9g), while the PDA/PPy/ND film remained stable without obvious damage. These results indicated that the introduction of PDA could improve the film-forming properties through both optimized dispersion in the PVA matrix and better water resistance. Actually, the PDA/PPy/ND hierarchical structures possessed better foregrounds for the potential application of electromagnetic absorption devices in extreme environments, such as high humidity. However, the MA performance of the film is a more complex issue which will be further studied in our future work.

## 4. Conclusions

A hierarchical mulberry-like PDA/PPy/ND hybrid was synthesized using in situ polymerization. It was found that PDA could egulate the morphology of the hybrids, synergistically improving microwave-absorption and flexible film-forming properties. The optimal RL peaks of PDA/PPy/ND could reach −43.6 dB at 7.58 GHz with a 3.3 GHz effective bandwidth. Flexible PDA/PPy/ND film could be obtained by using a simple solution-casting method, using PVA as a film-forming agent. The improvement in the microwave absorption is likely mainly due to the multiple dielectric losses resulting from the π-π stacking interactions and asymmetric polar hybridizations on the C-N groups, as well as the significantly improved impedance-matching characteristics caused by the hierarchical gradient structure. The PDA/PPy/ND hierarchical structure could be a potential material for electromagnetic absorption devices used in conditions of high humidity, which provides a referencing path for the practical application of lightweight electromagnetic-absorption materials.

## Figures and Tables

**Table 1:** The microwave-absorbing properties of some reported Ppy hybrids.

Samples	Optimal *RL* Peak, Operating Frequency	Effective Bandwidth	References
PDA/Ppy/ND	−43.6 dB, 7.58 GHz	3.3 GHz (6.2–9.5 GHz)	This work
Ppy/Fe_3_O_4_	−22.4 dB, 12.9 GHz	5.0 GHz (10.5–15.5 GHz)	[32]
Ppy/Fe_3_O_4_/GE	−40.53 dB, 6.32 GHz	5.12 GHz (11.12–16.24 GHz)	[35]
GPA(Ppy/GE)	−51.12 dB, 6.4 GHz	5.88 GHz (10.48–16.36 GHz)	[40]
C/Ni/Ppy	−42.09 dB, 13.26 GHz	5.24 GHz (10.4–15.6 GHz)	[41]
Ppy/CNFs/PDMS	−25 dB, 10.4 GHz	3.74 GHz (6.62–10.34 GHz)	[44]

## Data Availability

Not applicable.

## Supplementary Materials

The following supporting information can be downloaded at: https://www.mdpi.com/article/10.3390/polym14102014/s1, Figure S1: SEM images of (a) HCl-PPy, (b) PDA/PPy and (c) PDA/PPy/ND; Figure S2: The conductivities of the samples; Figure S3: The calculated (a) dielectric loss tangent (tan δe) and (b) magnetic loss tangent (tan δm) for the related samples; Figure S4: The photographs of the PPy/ND film without adding PDA.
