# Supplementary material for: Design of Flexible Film-Forming Polydopamine/Polypyrrole/Nanodiamond Hierarchical Structure for Broadband Microwave Absorption
Source: Polymers (Basel). 2022 May 15;14(10):2014. doi: 10.3390/polym14102014 (PMC9146107; doi:10.3390/polym14102014)
Supplement: Supplementary file 1 [file polymers-14-02014-s001.zip › polymers-1708216-supplementary.pdf]

Design of Flexible Film-Forming Polydopamine/ Polypyrrole/Nanodiamond Hierarchical Structure for Broadband Microwave Absorption

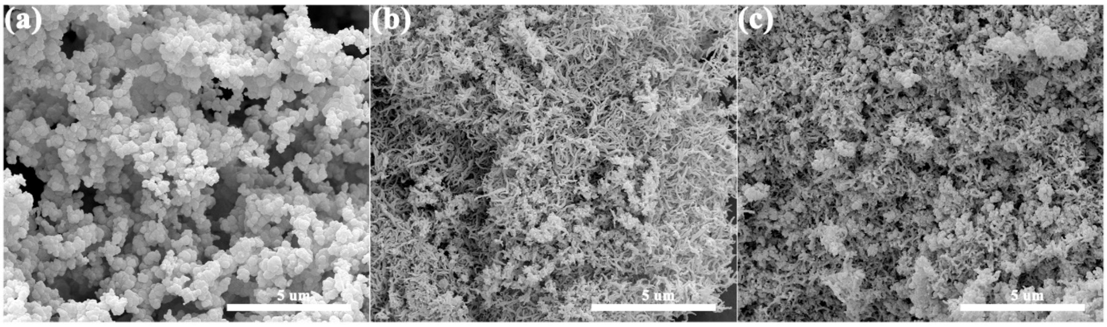

Figure S1. SEM images of (a) HCl-PPy, (b) PDA/PPy and (c) PDA/PPy/ND.

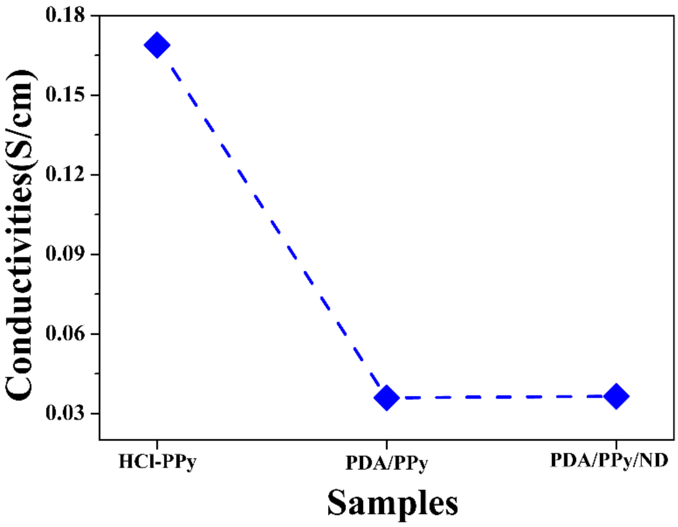

Figure S2. The conductivities of the samples.

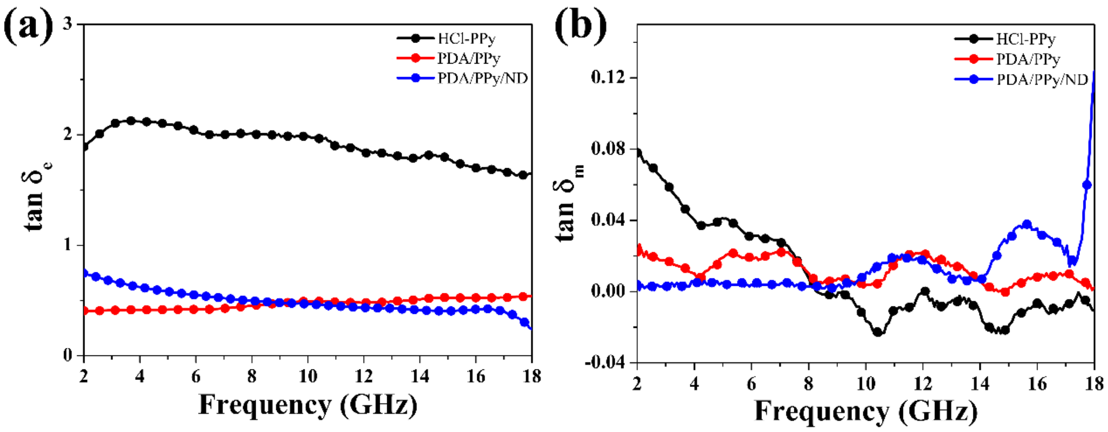

Figure S3. The calculated (a) dielectric loss tangent (tan δe) and (b) magnetic loss tangent (tan δm) for the related samples.

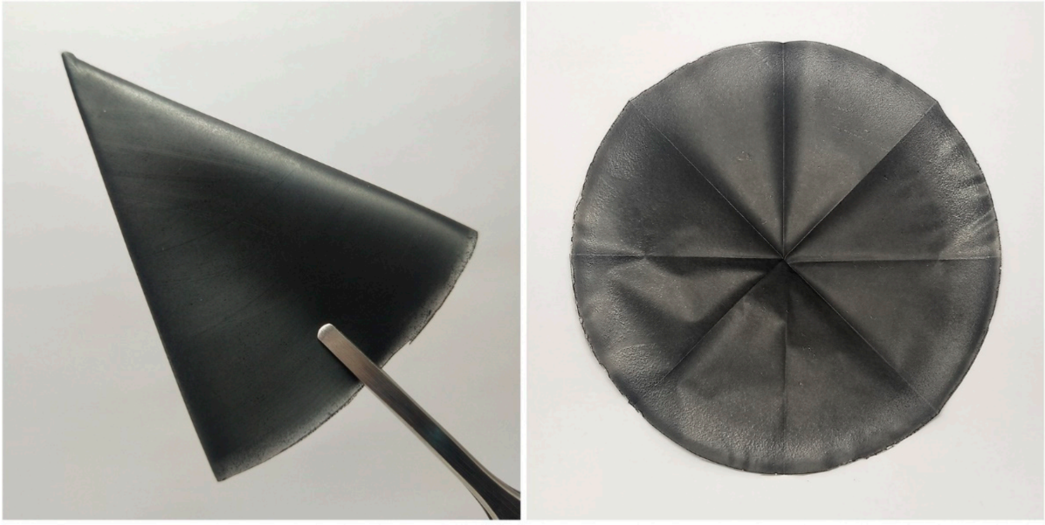

Figure S4. The photographs of the PPy/ND film without adding PDA.
